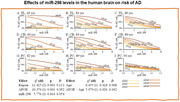# Elevated brain miR298 is associated with a reduced risk of Alzheimer’s disease

**DOI:** 10.1002/alz.089848

**Published:** 2025-01-03

**Authors:** Debomoy K. Lahiri, Ruizhi Wang, Bryan Maloney, Bernardino Ghetti, Andrew J. Saykin, Subodh Kumar, Fletcher A White, Nigel H Greig, Kumar Sambamurti, Scott E. Counts

**Affiliations:** ^1^ Indiana Alzheimer’s Disease Research Center, Indianapolis, IN USA; ^2^ Indiana University School of Medicine, Department of Psychiatry, Indianapolis, IN USA; ^3^ Indiana University School of Medicine, Department of Pathology & Laboratory Medicine, Indianapolis, IN USA; ^4^ Indiana Alzheimer's Disease Research Center, Indianapolis, IN USA; ^5^ Indiana University School of Medicine, Indianapolis, IN USA; ^6^ Texas Tech University Health Sciences Center El Paso, El Paso, TX USA; ^7^ Indiana University School of Medicine, Department of Anesthesia, Indianapolis, IN USA; ^8^ National Institute on Aging, NIH, Baltimore, MD USA; ^9^ Medical University of South Carolina, Department of Neurosciences, Charleston, SC USA; ^10^ Michigan Alzheimer’s Disease Research Center, Ann Arbor, MI USA; ^11^ Michigan State University, Grand Rapids, MI USA

## Abstract

**Background:**

Non‐coding RNA species, such as microRNA (miRNA), regulate multiple biological and pathological processes by binding to target mRNAs and facilitating alteration of translation levels via complexes such as RNA‐induced silencing complex (RISC). Disrupting this process could contribute to AD pathogenesis by fostering aggregation of hyperphosphorylated microtubule‐associated protein tau and amyloid‐β (Aβ) peptides, and neuroinflammation. Understanding how these pathological changes are regulated remains our research focus. We report that miR298 plays a vital role in maintaining APP and tau homeostasis and that miR298 imbalances may impact AD progression.

**Method:**

Levels of miR298 from non‐cognitively impaired (NCI) and AD subject brain tissue samples from different recognized sources were measured by qRT‐PCR and assessed for associations with AD risk and potential covariates such as age and APOE genotype. Other biomarkers were assessed in cortical samples from the same subjects, as we previously described. Further, APP, tau, and cytokines were profiled in miR298 mimic‐ or its antagomiR‐expressing human neuronal and astrocyte cultures.

**Result:**

Levels of miR298 varied in postmortem temporal lobe between AD patients and age‐matched NCI controls. Higher brain miR298 levels were associated with a reduced risk of AD. Subject age and APOE genotype altered this association; specifically, greater age and dose of the APOEε4 allele were associated with an increased risk of AD. However, APOEε4 dose‐associated risk reduced as age increased. We identified putative binding sites for miR298 on APP, BACE1, MAPT, IL1α, and IL6 mRNAs to form RISC. We showed that treatment by miR298 reduced tau, APP, and BACE1 proteins and mRNA levels in cell cultures.

**Conclusion:**

These studies suggest that miR298 regulates a coordinated network of AD‐related proteins APP, BACE1, and tau. Hence, such network regulation may represent a rational therapeutic target for reducing AD risk and disease modification. In addition to late‐onset cases, we will profile miR298 in brain tissue samples from early‐onset AD cases. Future work involves testing miR298 in AD animal models, such as in human tau‐overexpressing transgenic mice. We sincerely thank grant support from NIA/NIH.